# Immune correlates of XMRV infection

**DOI:** 10.1186/1742-4690-8-S1-A221

**Published:** 2011-06-06

**Authors:** Vincent Lombardi, Deborah Goetz, Max Pfost, Cassandra Puccinelli, Judy Mikovits

**Affiliations:** 1 Whittemore Peterson Institute, Reno, NV, 89557, USA; 2 Environmental Sciences,University of Nevada, Reno, NV, 89557, USA

## Background

CFS patients often display antiviral enzyme RNase L dysfunction underscoring the importance of the innate immune response in CFS. We reported the XMRV detection in the peripheral blood of 67% of a cohort of CFS patients and 3.4% of controls [1]. XMRV infection may play a role in CFS pathogenesis through the dysregulation of the immune response.

## Methods

This hypothesis was addressed by multiplex profiling of plasma cytokines and chemokines on a LuminexTM platform and phenotypic analysis of leukocyte subsets by multi-parameter flow cytometry in XMRV infected CFS patients versus uninfected controls. XMRV-infected subject and control samples were assayed blindly. Analysis was performed using the Gene Expression Pattern Analysis Suite and Random Forest tree classification algorithms. For immune profiling, 63 XMRV infected CFS patient samples were analyzed within 6 hours using an LSRII flow cytometer with BD FACSDiva software. Six normal donors and reference values based on a healthy population were used as normal baselines.

## Results

16 of the 26 cytokines/chemokines measured were significantly differentially expressed; eleven up-regulated and five down-regulated including: IL-8, IL-6, MIP1αlpha, MCP-1, IFNαlpha and TNFαlpha. XMRV-infected CFS patients showed reduced percentages of CD56+ NK and CD19+ B cells. The NK phenotype in XMRV-infected CFS patients was altered, with 80% of the patients having a significantly reduced CD56+DIM population. The B cells present in the peripheral blood were CD20+, CD23+ mature B cells.

## Conclusion

XMRV infection results in dysregulation of the immune response, either directly by infection of specific leukocyte subsets or indirectly through cytokine modulation.

## References

[B1] Lombardi V Ruscetti F Das Gupta J Pfost M Hagen K Peterson D Ruscetti S Bagni R Petrow-Sadowski C Gold B Dean M Silverman R Mikovits J Detection of an Infectious Retrovirus, XMRV, in Blood Cells of Patients with Chronic Fatigue Syndrome Science 2009 326 585 589 10.1126/science.1179052 19815723

